# An Unusual Suspect: Right Hypochondrium Pain in the Third Trimester of Pregnancy

**DOI:** 10.5334/jbsr.4193

**Published:** 2026-02-04

**Authors:** Dina Jleilati, Romain Gillard

**Affiliations:** 1 Intern in Radiology, Radiology Department, CHU de Liège, Belgium; 2 Radiologist, CHU de Liège, Beligum

**Keywords:** thrombosis, pregnancy, adrenal

## Abstract

*Teaching point:* Adrenal vein thrombosis should be considered in pregnant patients presenting with unexplained unilateral adrenal enlargement, even when the thrombus itself is not visualized, as early diagnosis allows effective anticoagulant management and prevention of adrenal insufficiency.

## Right Adrenal Vein Thrombosis in the Third Trimester of Pregnancy

### Case

**Figure 1:**
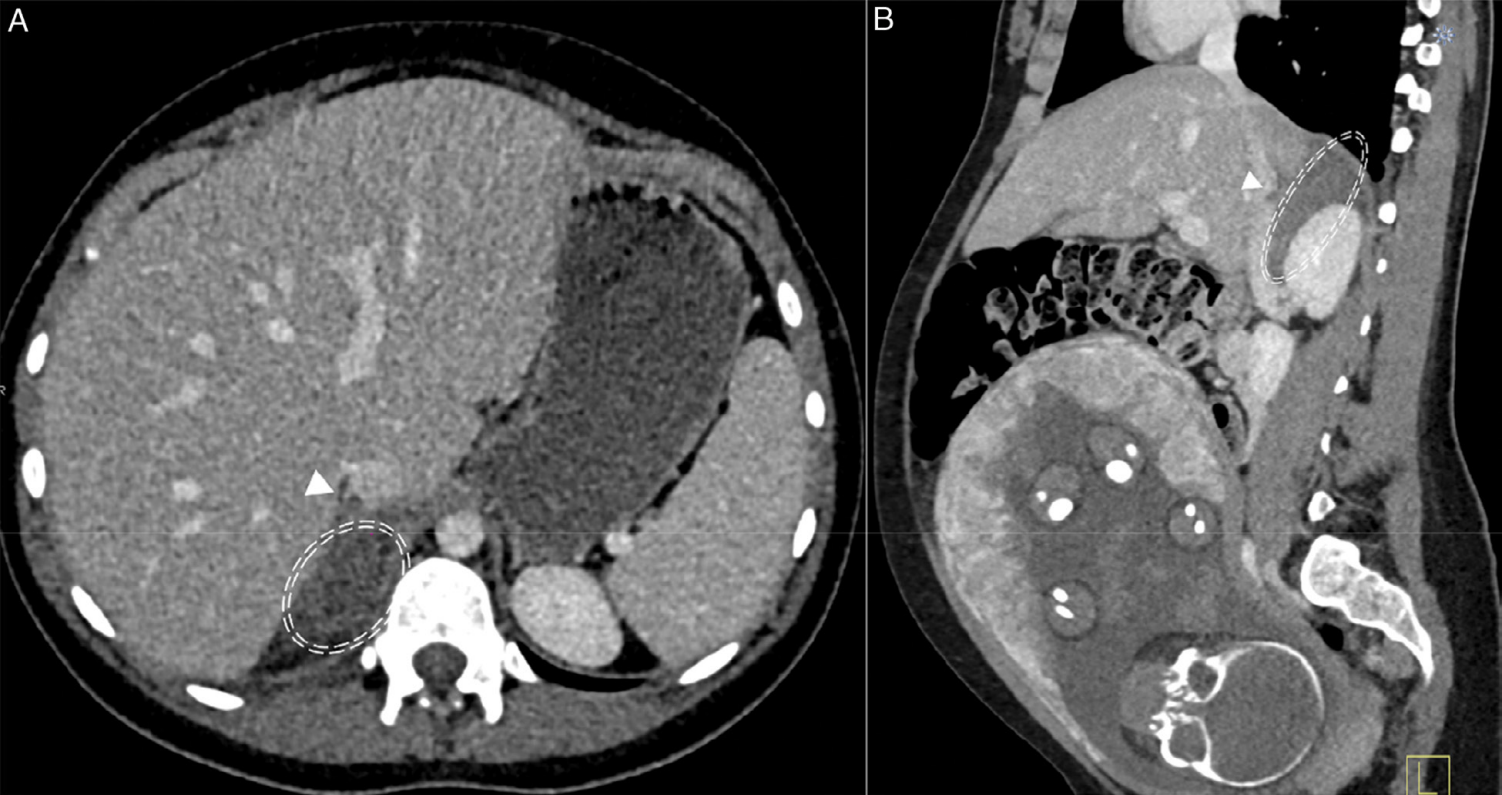
Axial **(A)** and sagittal **(B)** plane abdominal CT at portal phase showing poor enhancement and swollen appearance of the right adrenal gland (circle) and hypodense filling defect within the inferior vena cava (arrowhead), corresponding to the adrenal vein thrombosis overflowing in the inferior vena cava.

A 28-year-old woman at 32 weeks of pregnancy presented with acute right upper-quadrant abdominal pain radiating to the back. She had no urinary or gastrointestinal symptoms and was afebrile. Physical examination revealed localized right-sided tenderness without peritoneal signs. The obstetric assessment was normal. Laboratory tests showed elevated CRP and moderate leukocytosis. An initial ultrasound revealed no abnormalities. Because the pain persisted and inflammatory markers remained elevated, a contrast-enhanced CT scan was performed. The CT demonstrated an enlarged hypodense right adrenal gland with periadrenal fat stranding. A filling defect extending from the right adrenal vein into the inferior vena cava (IVC) was identified, confirming the diagnosis of right adrenal vein thrombosis. Figures 1A and 1B show an axial (a) and sagittal (b) plane abdominal CT at portal phase showing poor enhancement and swollen appearance of the right adrenal gland (circle) and hypodense filling defect within the IVC (arrowhead), corresponding to the adrenal vein thrombosis overflowing in the IVC. Therapeutic anticoagulation with enoxaparin was initiated, resulting in progressive resolution of symptoms.

Postpartum thrombophilia screening demonstrated persistently elevated factor VIII levels and a heterozygous G20210A prothrombin gene mutation, supporting an underlying inherited thrombophilia. Anticoagulation was continued for six weeks postpartum.

### Comment

Adrenal vein thrombosis is a rare but clinically significant cause of acute abdominal pain, particularly in pregnant patients in the third trimester. Its presentation is often non-specific and overlaps with several more common differentials, making timely diagnosis challenging.

Its true incidence is likely underestimated because imaging is required for diagnosis, and the condition may be misattributed to more common causes of abdominal pain in pregnancy. The right adrenal gland is disproportionately affected owing to its direct drainage into the IVC and the increased venous stasis induced by uterine compression during late gestation. Progression to adrenal insufficiency is rare because bilateral involvement is exceptional [1].

Imaging plays a key diagnostic role. The thrombus is often not directly visualized; therefore, the diagnosis relies on characteristic indirect signs. CT typically shows unilateral adrenal enlargement, parenchymal hypoattenuation, and periadrenal fat stranding. When visible, a filling defect in the adrenal vein or IVC confirms thrombosis (Figures 1A and 1B). MRI can be used to avoid fetal radiation and may demonstrate findings consistent with edema, infarction, or hemorrhage [1].

Awareness of this diagnosis in cases of unexplained adrenal swelling is essential, even in the absence of direct thrombus visualization. It helps ensure prompt management and good maternal-fetal outcomes.
